# Mr. Haines, on Preserving Vaccine Virus

**Published:** 1803-07-01

**Authors:** W. Haines

**Affiliations:** Hambledon


### *To the Editors of the Medical and Physical Journal.*

Gentlemen,

So many methods having been proposed for preserving the Vaccine Virus, that practitioners appear undetermined which to adopt; and as your valuable publication holds out the best channel for communicating the result of experience in the different modes, I beg leave to offer my testimony in favour of that described by Mr. Dennett, in a former number of your Journal. A similar one was lately offered to notice in the same work, which appears not to possess any superior advantage, and partakes less of the simplicity of the former.

I have at different times received quills from Mr. Dennett, for myself and others, my medical friends, and have found that the virus thus kept, to answer more decidedly than any other which I have seen noticed. As this is a matter of great importance to practitioners, particularly those who practice at a distance from London, I request for it a place in the Medical and Physical Journal.

I am, &c.

